# IMPACT OF COVID-19 ON OCCUPATIONAL INJURY AMONG LONG-TERM CARE WORKERS IN CALIFORNIA

**DOI:** 10.1093/geroni/igac059.627

**Published:** 2022-12-20

**Authors:** Soo-Jeong Lee

**Affiliations:** University of California, San Francisco, San Francisco, California, United States

## Abstract

Long-term care is the industry with the highest occupational injury and illness rate in the United States. During the Coronavirus Disease 2019 (COVID-19) Pandemic, healthcare workers have been affected physically and psychologically by increased workload and stress. Such increased physical and psychological demands may increase the risk of injury among long-term care workers. The aim of this study is to assess the impact of COVID-19 on occupational injury or illness incidence among long-term care workers. Analyzing California Workers’ Compensation (WC) data reported in 2019 and 2020, the study will report the number and rate of WC claims among California long-term care workers by month and examine changes over time during 2019-2020. The study will also characterize WC claims during 2019-2020 by demographic (age, gender), occupational (occupation, job tenure), and injury or illness characteristics (cause of injury, nature of injury, affected body part) and identify characteristics with greater changes during COVID-19.

